# Supplementary figures and images for: Comparative Transcriptome Analysis Reveals the Effect of Lignin on Storage Roots Formation in Two Sweetpotato (Ipomoea batatas (L.) Lam.) Cultivars
Source: Genes (Basel). 2023 Jun 14;14(6):1263. doi: 10.3390/genes14061263 (PMC10297969; doi:10.3390/genes14061263)

# Pearson correlation between samples

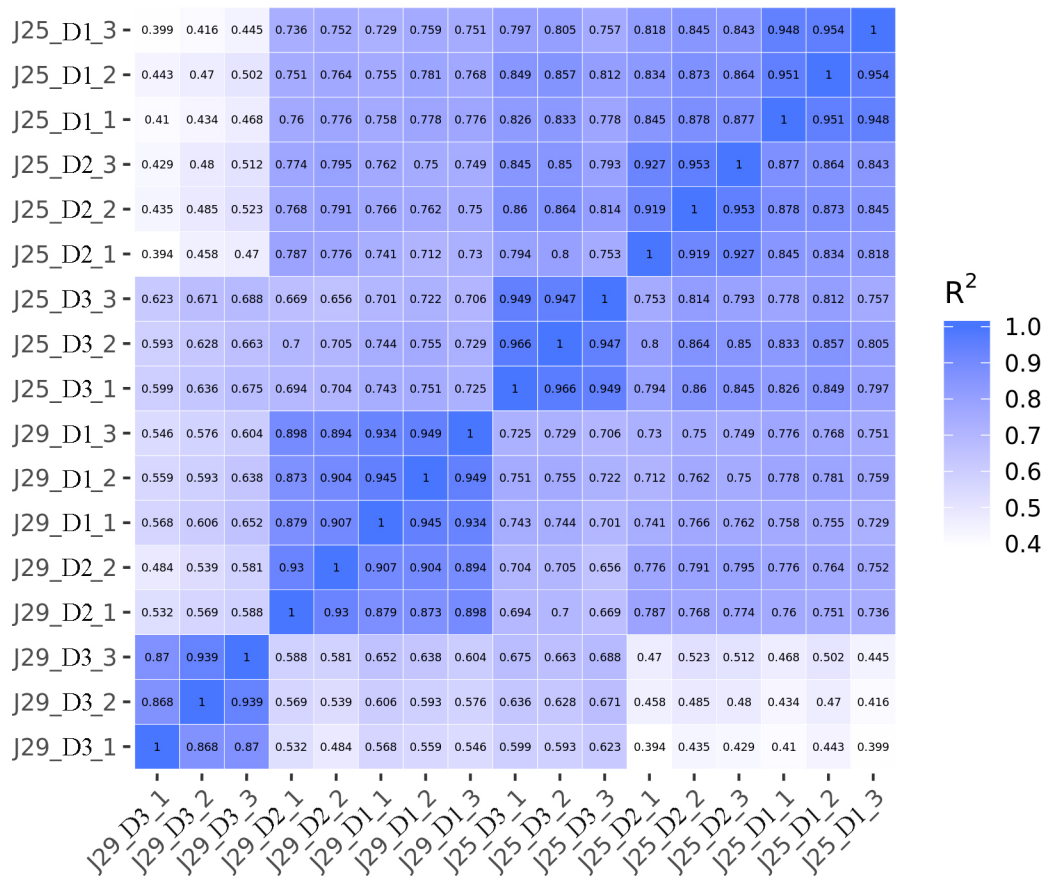

Supplement: Supplementary file 1 [file genes-14-01263-s001.zip › Figure S1.pdf]

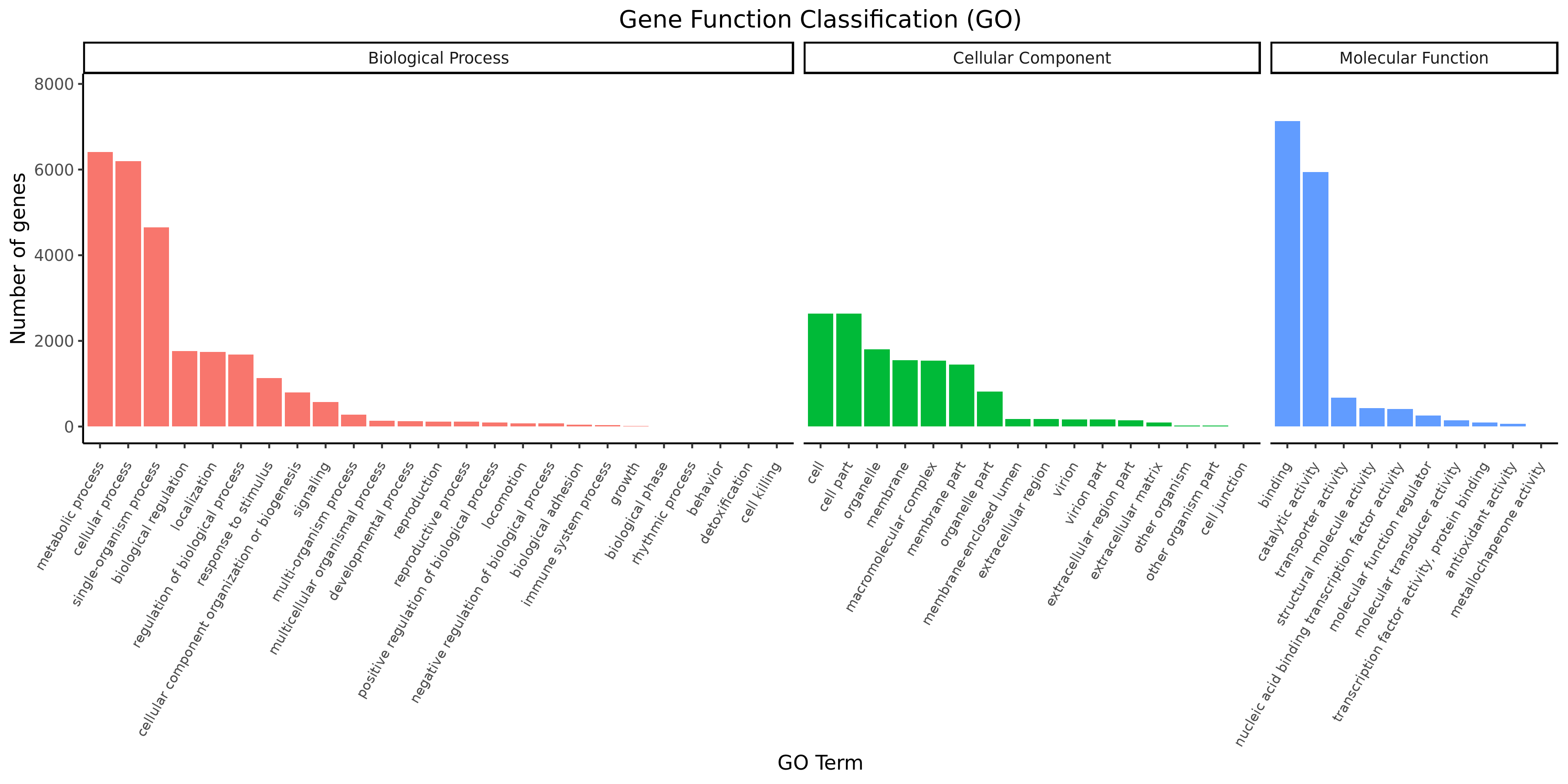

Supplement: Supplementary file 1 [file genes-14-01263-s001.zip › Figure S2.png]

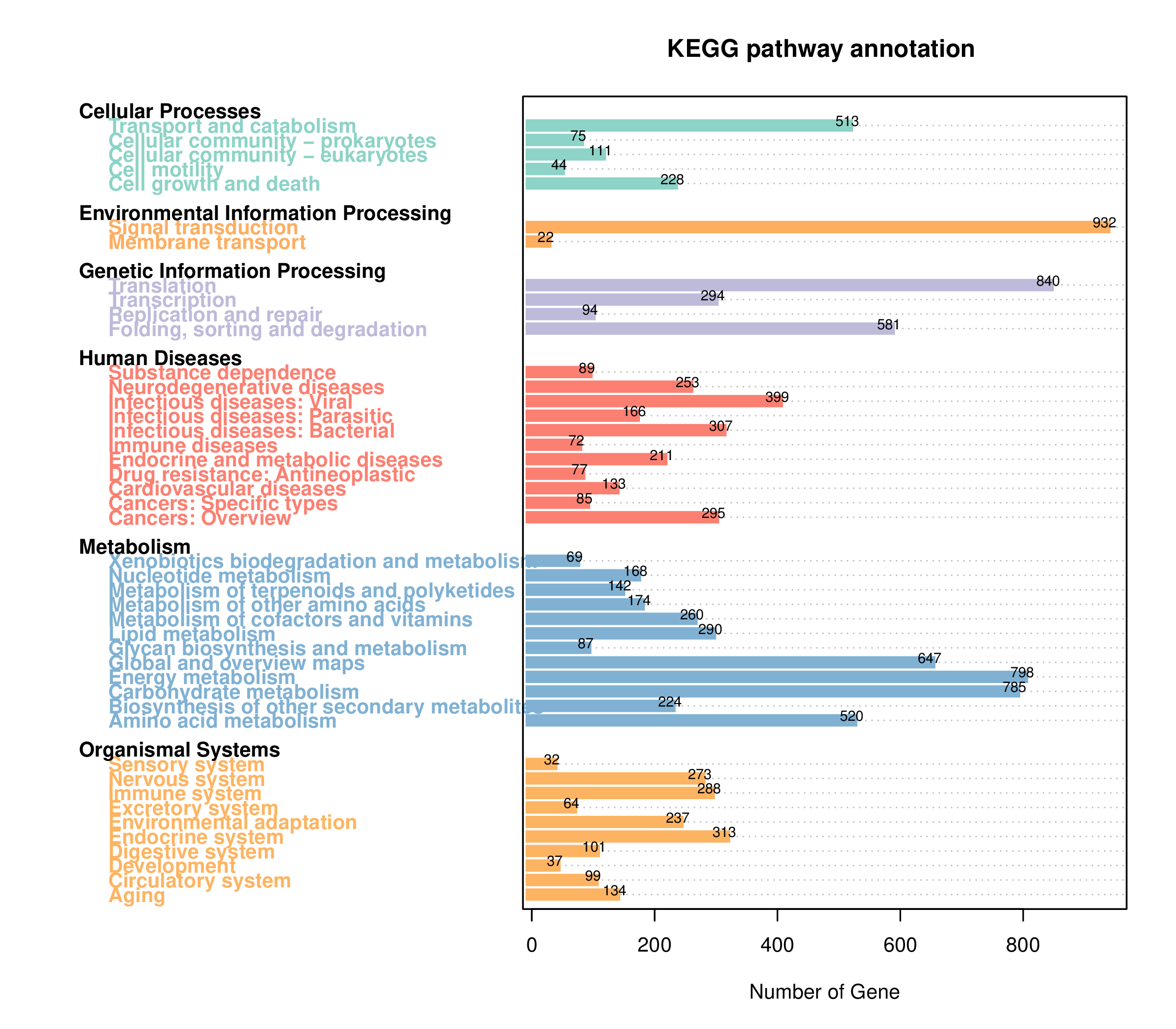

Supplement: Supplementary file 1 [file genes-14-01263-s001.zip › Figure S3.png]

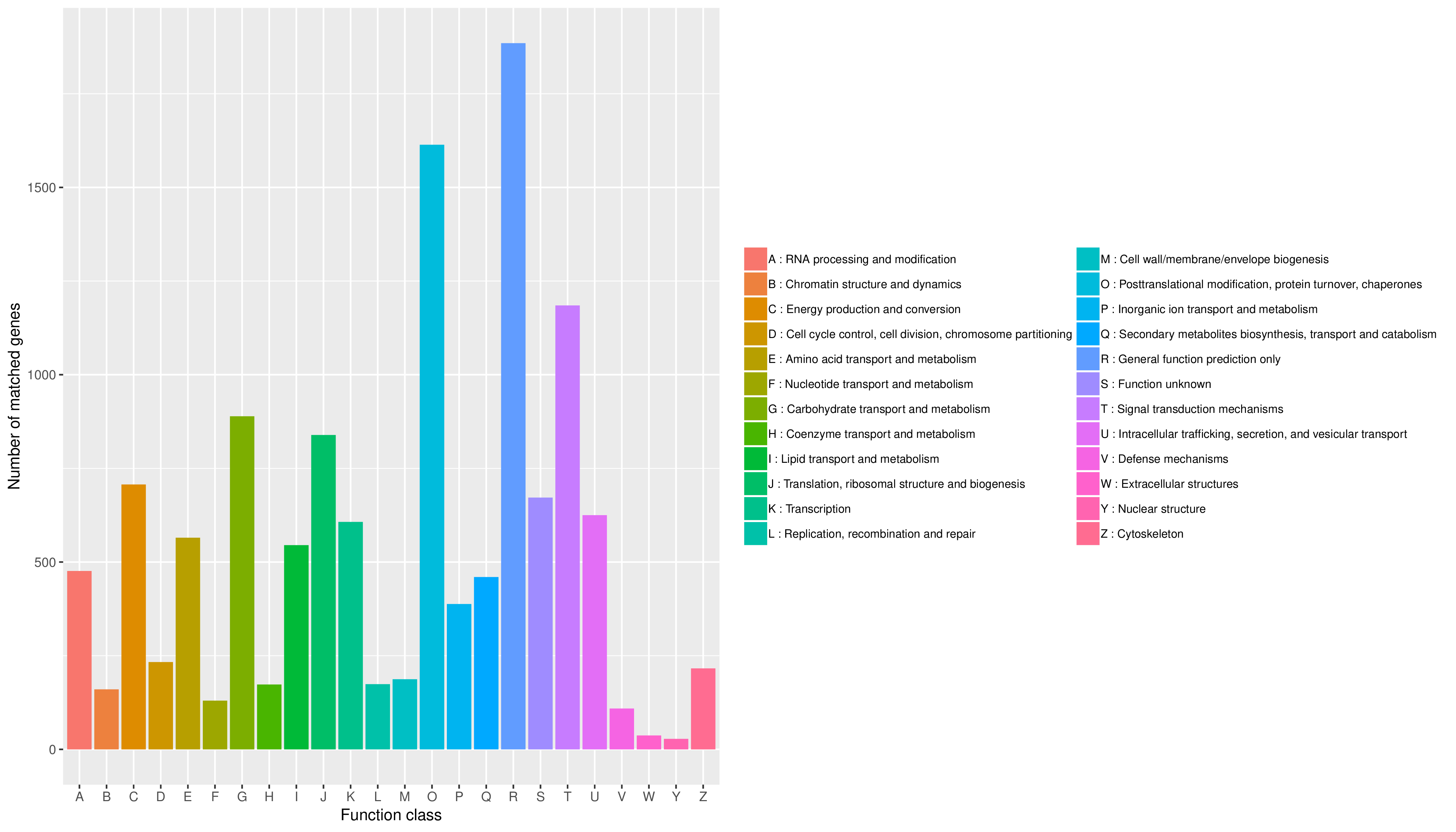

Supplement: Supplementary file 1 [file genes-14-01263-s001.zip › Figure S4.png]
